# SARS-CoV-2 Seroprevalence Post-First Wave among Primary Care Physicians in Catania (Italy)

**DOI:** 10.3390/tropicalmed6010021

**Published:** 2021-02-09

**Authors:** Caterina Ledda, Flavia Carrasi, Maria Teresa Longombardo, Gianluca Paravizzini, Venerando Rapisarda

**Affiliations:** 1 Occupational Medicine, Department of Clinical and Experimental Medicine, University of Catania, 95100 Catania, Italy; L98002367@studium.unict.it (F.C.); vrapisarda@unict.it (V.R.); 2 Clinical Laboratories “Girlando and Paravizzini”, 95100 Catania, Italy; m.longombardo@girlandoeparavizzini.com (M.T.L.); gianluca@paravizzini.net (G.P.)

**Keywords:** COVID-19, healthcare worker, healthcare personnel, SARS-CoV-2, general practitioners, medical doctor

## Abstract

Family physicians or pediatricians and general practitioners (GPs) work in non-hospital settings. GPs usually visit many patients, frequently at their homes, with low potential, if any, to control the work setting. Particularly during the initial phases of the COVID-19 outbreak, they were not informed about the occurrence of SARS-CoV-2-infected patients, with inadequate information regarding the risk, a lack of suitable protective measures and, in some cases, deficient or poor accessibility to personal protective equipment (PPE). During the first wave of COVID-19, primary care physicians were on the front line and isolated the first cases of the disease. The present study aims to estimate the seroprevalence of SARS-CoV-2 in a cohort of 133 GPs working in Catania (Italy) after the first wave of COVID-19. Serological analysis revealed a low seroprevalence (3%) among GPs. The low seroprevalence highlighted in the results can be attributed to correct management of patients by GPs in the first wave. It is now hoped that mass vaccination, combined with appropriate behavior and use of PPE, can help further reduce the risk of COVID-19 disease.

## 1. Introduction

The novel coronavirus (SARS-CoV-2), the causative agent of Corona Virus Disease-2019 (COVID-19), is the most severe global public health emergency of the twenty-first century [1].

The disease, which appeared in Wuhan, China, in December 2019, quickly spread across continents and was declared a pandemic by the World Health Organization (WHO) on 11 March 2020 [2].

At 5 February 2021, SARS-CoV-2 has infected over 104,000,000 persons and caused over 2,200,000 deaths globally [2].

Current information suggests that, in community settings, person-to-person transmission most commonly occurs via the respiratory droplets of the infected individual (through coughing, sneezing, talking, etc.), close interaction with the infected person, or self-delivery of the microorganism to the eyes, nose, or mouth via SARS-CoV-2 contaminated hands [3].

In health care scenarios, in addition to respiratory droplet-borne or contact-borne transmission, airborne transmission can also occur via aerosol-generating operations [3].

SARS-CoV-2 is highly transmissible, and health care workers (HCWs) have been occupied with the important risk of providing care to supposed or confirmed COVID-19 cases. Some reports have shown that numerous HCWs have contracted COVID-19 in health care settings globally [4,5,6,7,8].

The WHO, the Center for Disease Control and Prevention (CDC), and the European Centre for Disease Prevention and Control (ECDC) issued procedures for COVID-19 management that endorse safety protocols for HCWs, such as using appropriate personal protective equipment (PPE).

Nevertheless, because the suggested infection control protections have not been adequate to prevent the spread of the SARS-CoV-2 infection among HCWs, unrecognized risk factors may contribute to the transmission of viruses in hospitals [4,5,6,7,8].

However, not all healthcare is provided in a hospital setting. In particular, family physicians or pediatricians and general practitioners (GPs) work in a setting away from the hospital.

GPs usually visit numerous patients, frequently at their homes, with low potential, if any, to control the work setting. Particularly during the initial phases of the COVID-19 outbreak, GPs were not informed of the occurrence of SARS-CoV-2-infected patients, with inadequate information about the risk, a lack of suitable protective measures and, in some cases, deficient or poor accessibility to PPE [9].

Modenese and Gobba reported that 44% of the COVID-19 related deaths among Italian physicians were GPs [10].

In Italy, the first wave started at the end of January 2020 and in March, the government implemented a series of restrictive measures. On March 5th, all schools and universities in Italy locked their buildings to scholars. From 8 March to 4 May, the population was forced to respect the lockdown.

In the province of Catania, 777 cases [11] of COVID-19 were reported until the end of June, that is, the period of the effect of the lockdown.

The present study aims to estimate the seroprevalence of SARS-CoV-2 in a cohort of GPs working in Catania (Italy) after the first wave of COVID-19.

## 2. Materials and Methods

In the province of Catania, there are approximately 1100 working GPs. From June to July 2020, GPs working in the province of Catania (Italy) were recruited in this study through contacts made via mail from a GP database obtained from the Medical Board.

The GPs who made themselves available to participate in the study were contacted by email and filled out an online form that included the registration of: personal and work data, number of patients with COVID-19, any previous diagnosis of COVID-19 through molecular swab, eventual close contact with cases of COVID-19, possible presence of symptoms of COVID-19 attributable to contact, presence of other pathologies.

Following the receipt of answers from the GPs, blood sampling sessions were organized, two at the University of Occupational Medicine, and four in the province. The collected serum was immediately taken to the laboratory and analyzed the same day.

Serological analysis was performed using The NovaLisa^®^ SARS-CoV-2 IgG (NovaTec Immundiagnostics GmbH, Dietzenbach, Germany) ELISA method. The aim of the test was the qualitative detection of IgG antibodies to SARS-CoV-2 virus in human serum.

Informed consent was obtained from all GPs. The present study was approved by the ethics committee of Catania University Hospital (n. 54/2020).

Data were analyzed with the software SPSS 22.0 (SPSS Inc., Chicago, IL, USA) for Windows. Descriptive analyses were performed using frequency percentages and mean and standard deviation.

## 3. Results

An invitation email was sent to 170 GPs. A total of 133 elected to participate in the study (response rate: 78%). The characteristics of the participating GPs are detailed in Table 1.

As reported in Table 1, three GPs had a COVID-19 diagnosis. SARS-CoV-2 antibodies were detected in 4 of 133 (3%) of GPs, and 2 (2%) had a “borderline” result. Table 2 reports the seroprevalence results after ELISA analysis.

## 4. Discussion

The aim of this study was to assess the SARS-CoV-2 seroprevalence, after the first wave of the disease, among GPs working in Catania province (Italy). Probable cases, according to WHO criteria [12], were 21 (20%). Serological analysis revealed a low seroprevalence (3%) among GPs.

These low seroprevalence rates were found despite the treatment of 777 patients with COVID-19 infections during the first wave [11] period in which GPs worked with very few PPE available from the local health authority. Nevertheless, the degree of contagion was lower in GPs compared to HCWs in direct contact with COVID-19 patients in hospital settings. Other seroprevalence studies carried out among HCWs working in hospital report higher rates of prevalence: Belgium (12.6%) [13], Spain (11.2%) [14], Italy (14.4%) [15], Sweden (19.1%) [16], the United Kingdom (10.8–43.5%) [13,17], and the United States of America (7.6–13.7%) [18,19]. The differences in these rates may be due to differences in seroprevalence in the general population by May 2020: 8.0% in Belgium, 5.5% in Spain, 4.6% in Italy, 3.7% in Sweden, 5.1% in the United Kingdom, and only 0.85% in Germany [20].

A recent study on duration of SARS-CoV-2 antibody carried out by Lumley et al. [21] evidences that anti-spike IgG levels remained stably detected after a positive result in 94% of health care workers after 180 days, instead anti-nucleocapsid IgG levels rose to a peak at 24 and the mean estimated antibody half-life was 85 days. Ongoing longitudinal studies are required to track the long-term duration of antibody levels and their association with immunity to SARS-CoV-2 reinfection and evaluate these two IgG fractions for guide vaccination modalities.

To date, no seroprevalence studies have been carried out among GPs by other research groups.

Due to the type of work and the shortage of PPE, GPs contracted the virus at the beginning of the spread of SARS-CoV-2. Subsequently, distancing rules were also adopted within clinics, and overnight stays and contact were avoided unless requested. Thus, contagion was able to be reduced.

It is has been found that rigorous use of appropriate PPE by HCWs when providing direct care of all patients efficiently prevents COVID-19 transmission [22,23]. Moreover, a study carried out by Brehm underlines that mindfulness of COVID-19 is crucial even when suitable PPE is used.

Our results are in line with an investigation carried out by Arons [24], which proved that pre-symptomatic patients play a crucial role in the spread of SARS-CoV-2 associated with both community and healthcare facilities.

It is important to finalize strategies and procedures to prevent SARS-CoV-2 infection among GPs, for example, specific guidelines for GPs to safely guarantee care for patients during the spread of COVID-19. Another input could be the introduction of telemedicine procedures to reduce direct contact with patients [10,25,26,27].

In conclusion, the low seroprevalence highlighted in these results can be attributed to correct management of patients by GPs during the first wave. It is now hoped that mass vaccination, in combination with appropriate behavior and use of PPE, can help further reduce the risk of COVID-19 disease.

## Figures and Tables

**Table 1:** Characteristics of participating general practitioners (GPs).

Variables	Results
Gender (Male; n, %)	62 (47%)
Age (Mean ± SD)	55.65 ± 11.24
Working seniority (Mean ± SD)	15.14 ± 13.62
Patients	994.44 ± 358.33
GPs with COVID-19 patients (n., %)	51 (38%)
GPs who had swabbed for SARS-CoV-2 RT detection (n., %)	21 (16%)
GPs with COVID-19 diagnosis	3 (2%)
GPs with close contacts with cases of COVID-19 (n., %)	78 (59%)
Probable cases	21 (20%)
Presence of COVID-19 symptoms (n., %)	
Hyposmia * (n., %)	4 (3%)
Ageusia * (n., %)	2 (2%)
Fever > 37.5 °C * (n., %)	23 (23%)
Fatigue * (n., %)	65 (49%)
Muscle aches * (n., %)	52 (52%)
Sore throat * (n., %)	50 (50%)
Dry cough * (n., %)	48 (36%)
Nasal congestion * (n., %)	41 (31%)
Rhinorrhea * (n., %)	40 (30%)
Dyspnea * (n., %)	9 (7%)
Headache * (n., %)	53 (40%)
Abdominal pain * (n., %)	23 (17%)

* More than one answers was given.

**Table 2:** Seroprevalence of GPs.

Results	n. (%)
Negative	127 (95%)
Positive	4 (3%)
Borderline	2 (2%)

## Data Availability

Data available on request due to privacy restrictions.
